# Epigenetics Decouples Mutational from Environmental Robustness. Did It Also Facilitate Multicellularity?

**DOI:** 10.1371/journal.pcbi.1003450

**Published:** 2014-03-06

**Authors:** Saurabh Gombar, Thomas MacCarthy, Aviv Bergman

**Affiliations:** 1 Department of Systems and Computational Biology, Albert Einstein College of Medicine, Bronx, New York, New York, United States of America; 2 Department of Applied Mathematics and Statistics, State University of New York, Stony Brook, New York, United States of America; University of Washington, United States of America

## Abstract

The evolution of ever increasing complex life forms has required innovations at the molecular level in order to overcome existing barriers. For example, evolving processes for cell differentiation, such as epigenetic mechanisms, facilitated the transition to multicellularity. At the same time, studies using gene regulatory network models, and corroborated in single-celled model organisms, have shown that mutational robustness and environmental robustness are correlated. Such correlation may constitute a barrier to the evolution of multicellularity since cell differentiation requires sensitivity to cues in the internal environment during development. To investigate how this barrier might be overcome, we used a gene regulatory network model which includes epigenetic control based on the mechanism of histone modification via Polycomb Group Proteins, which evolved in tandem with the transition to multicellularity. Incorporating the Polycomb mechanism allowed decoupling of mutational and environmental robustness, thus allowing the system to be simultaneously robust to mutations while increasing sensitivity to the environment. In turn, this decoupling facilitated cell differentiation which we tested by evaluating the capacity of the system for producing novel output states in response to altered initial conditions. In the absence of the Polycomb mechanism, the system was frequently incapable of adding new states, whereas with the Polycomb mechanism successful addition of new states was nearly certain. The Polycomb mechanism, which dynamically reshapes the network structure during development as a function of expression dynamics, decouples mutational and environmental robustness, thus providing a necessary step in the evolution of multicellularity.

## Author Summary

Understanding the transition to multicellularity remains a key unanswered question in evolutionary biology. The transition required three essential cellular features to evolve: adhesion, signaling and differentiation. In particular, cell differentiation requires sensitivity to environmental cues to create distinct cell-specific transcription profiles. Previous work with model organisms and gene network models showed that biological systems evolve robustness to both mutational and environmental perturbations under stabilizing selection and that furthermore, mutational and environmental robustness are correlated. Increased robustness to environmental cues will therefore pose a barrier to the development of cell differentiation, and thus multicellularity. Because several important epigenetic developmental mechanisms, particularly Polycomb-mediated histone modification, appear to have evolved with multicellularity, we hypothesized that such a mechanism facilitated sensitivity to the environment and therefore cell differentiation. Using a computational model, we integrated Polycomb function with a regulatory model, revealing a clear decoupling between environmental and mutational robustness, allowing increased environmental sensitivity while mutational robustness remained intact. We also found that Polycomb greatly facilitated the ability for a single gene network to create several distinct transcription profiles - each representing a distinct differentiated cell type. Our work highlights the simple elegance through which the evolution of a key epigenetic mechanism can facilitate the transition to functional cell differentiation.

## Introduction

Understanding the evolution of major transitions in the complexity of organisms remains one of the key challenges in modern biology [1], [2]. In particular, the transition to multicellularity required the evolution of several innovations at the molecular level in order to satisfy three key requirements: cell-to-cell adhesion, cell-to-cell signaling, and cellular differentiation [3], [4]. Such molecular innovations can often be facilitated by genomic duplication and subsequent specialization [5] as well as other evolutionary processes such as exaptation [6], [7] and coevolution [8]. In the case of cellular differentiation, the evolution of epigenetic gene regulation is arguably the most important; enabling molecular innovation during the expansion of the Metazoa [9], [10]. Of course, molecular innovations are also subject to multiple constraints which may be imposed externally through the environment [11] or internally, for example as a consequence of the developmental process [12]. Here we will be concerned with robustness as an evolved internal constraint.

Robustness in biological systems is the property of persistent behavior despite genetic and environmental insults. Previous studies, using gene regulatory network models, have shown that networks will evolve robustness to genetic mutations under conditions of stabilizing selection [13], [14]. This result has been experimentally verified in RNA viruses [15], yeast [16], [17], and in the process of RNA folding [18]. In addition to genetic mutations, organisms are exposed to environmental changes. Previous studies using gene regulatory network models have shown that environmental and mutational robustness are positively correlated and are therefore expected to increase together under stabilizing selection [16], [17], [18], [19], [20], [21]. Furthermore, studies exploring robustness of miRNA sequence have shown that mutational robustness develops directly in response to evolving environmental robustness [22]. Indeed computational models of cell differentiation also show the presence of robustness [23]. However, invariance to the environment poses an obstruction to cell differentiation in multicellular organisms where internal environmental factors dictate cell fate decisions. Highlighting the Metazoan cell differentiation dependence on the environment is recent work showing that changes in a small number of key growth factors is capable of altering cell fate decisions [24], [25]. For example, changes in expression of *ct4*, *Sox2*, *Klf4* and *c-Myc* can drive conversion of fibroblasts to cardiomyocytes [26]). Furthermore, the developmental impact of environmental sensitivity can be observed in the developing human fetus which is most vulnerable to environmental chemicals such as alcohol within the first few weeks of pregnancy [27], [28], [29]. Therefore, how did multicellular organisms develop sensitivity to the internal environment, promoting cell differentiation, while retaining mutational robustness?

The available evidence suggests that the transition to multicellularity was accompanied by major innovations in epigenetic regulation [30], [31], [32]. Indeed chromatin states are in large part responsible for the gene expression differences across cell types [33], [34], [35], [36]. Post-translational modification of histones alters chromatin structure to encourage or repress transcription. A key group of proteins responsible for marking regions for transcriptional repression during development are the Polycomb Group Proteins (PcGs). Early studies elucidated the general functionality of this protein group in developing *Drosophila* embryos. In particular it was found that the chromosomal regions targeted by PcGs were transcriptionally repressed only if genes in the region were exhibiting low levels of expression when the PcGs became active [37]. In this manner the PcGs were found to be responsible for turning off discrete sets of genes in different cell types depending on expression levels during early development. For example, *MyoD*, a transcription factor required for myogenic commitment, is unable to access its binding sites in non-myoblast cells due to PcG dependent methylation [38]. In addition, it has been shown that activation of muscle-specific genes in the vicinity of the PcG binding site prevent the PcGs from hypermethylating the site, thus allowing MyoD to exert transcriptional activation effects. This functionality has motivated speculation that PcGs may have aided in the transition from a unicellular to a multicellular world by promoting differential expression in cell differentiation [39], [40]. Supporting this hypothesis, evolutionary analysis of the PcG Polycomb Repressive Complex 2 (PCR2) has revealed that homologs of the core components (E(Z), ESC, Su(z)12, and Nurf55) existed prior to multicellular lineages but were rarely found present as a functional complex in single cell organisms (although it is likely the last common unicellular ancestor of Metazoa did have all the components in place) [39], [40], [41]. In addition, *Saccharomyces cerevisiae* and other unicellular fungi with multicellular ancestors do not have the full set of functional homologs, correlating the loss of PcGs with reversal of multicellularity [39].

To explore how a dynamic epigenetic process such as chromatin modification affects robustness and cell differentiation we have extended a well-established gene regulatory network model [13], [42] with an epigenetic mechanism modeled on the Polycomb system. In accordance with previous results we find that in the absence of an epigenetic mechanism both mutational and environmental robustness co-evolve by increasing together. However, with the introduction of the Polycomb mechanism we see a decoupling of environmental and mutational robustness. Mutational robustness still increases under stabilizing selection in concordance with experimental results but environmental robustness decreases, thus increasing responsiveness to the environmental cues. In order to evaluate the capacity for cell differentiation in the model, we quantified the ability for producing alternative steady states (outputs) in response to novel environmental conditions (inputs). Consistent with the increase in environmental sensitivity we found that the Polycomb mechanism greatly facilitated the ability to create new input/output mappings, suggesting a strongly increased capacity for generating alternative cell fates. Our results suggest a clear link between epigenetic regulation and cell differentiation in that the epigenetic mechanism allows a gene regulatory network to be altered dynamically, effectively creating multiple networks out of a single regulatory architecture.

## Results

### Description of the model

In order to study the evolution of a Polycomb-like epigenetic mechanism we extended an established model of evolution in gene regulatory networks [13], [42]. Briefly (see Methods for details), the model functions on two levels: population dynamics and gene regulatory network (the genotype-phenotype mapping). At the lower level of the genotype-phenotype mapping, the genotype of each individual is represented as a gene regulatory network of 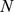 genes. Gene expression dynamics are initiated by an input vector 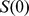, leading to a steady state 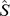 of length 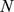 this defines the phenotype (individuals not reaching steady state have zero fitness). At the population dynamics level individuals undergo iterations of mutation, reproduction and selection. We measure mutational robustness as described previously [13], [14], [43] by randomly mutating an entry in the interaction matrix 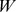 (of size 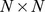) and comparing the effect on the phenotype 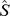 to that for the unmutated 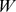 matrix. Following Ciliberti et al [19], we measure environmental robustness by introducing random changes into the input vector 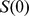 and similarly considering the effect on the phenotype 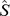.

Epigenetic regulation through chromatin remodeling is postulated to be a key mechanism through which a single genome can dynamically change gene expression to produce distinct stable cell types [30], [31], [32]. To determine the effect of epigenetic mechanisms on the two distinct forms of robustness we incorporated Polycomb group (PcG)-like activity into the gene regulatory model. Here, we assume that Polycomb is expressed beginning at time 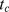 during development. Susceptibility to Polycomb for each gene 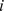 (representing the presence of *cis*-acting Polycomb Response Elements) is determined by 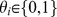 such that from time 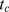 onwards, the expression of each gene is repressed by the Polycomb protein if 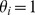 and its expression level falls below a threshold level 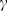. This behavior is modeled upon the known function of the Polycomb Repression Complex 1 (*PRC1*) in the *Drosophila* embryo where the Hox genes (whose initial expression is determined by transiently expressed Gap genes) are permanently repressed by PRC1, thus maintaining anterior/posterior expression patterning [44]. More formally the expression dynamics are defined by:
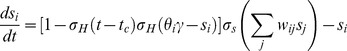
(1)Where 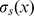 is the sigmoid function defined as 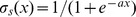 and 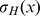 is a Heavy-side function that equals 0 if x<0 and 1 if x≥0. Susceptibility to Polycomb for each gene is set to 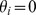 for all genes at the beginning of each simulation (generation 0) but is subject to change at a mutation rate 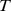 such that genes can gain or lose susceptibility (i.e. the variable 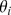 transitions between 0 and 1 with probability 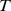 in each offspring). Here we are modeling the evolution of the Polycomb Response Element (PRE), a small canonical base sequence that is targeted by PcGs in higher metazoans [45], [46].

### Evolution with Polycomb mechanism decouples environmental and mutational robustness

**Figure 1:**
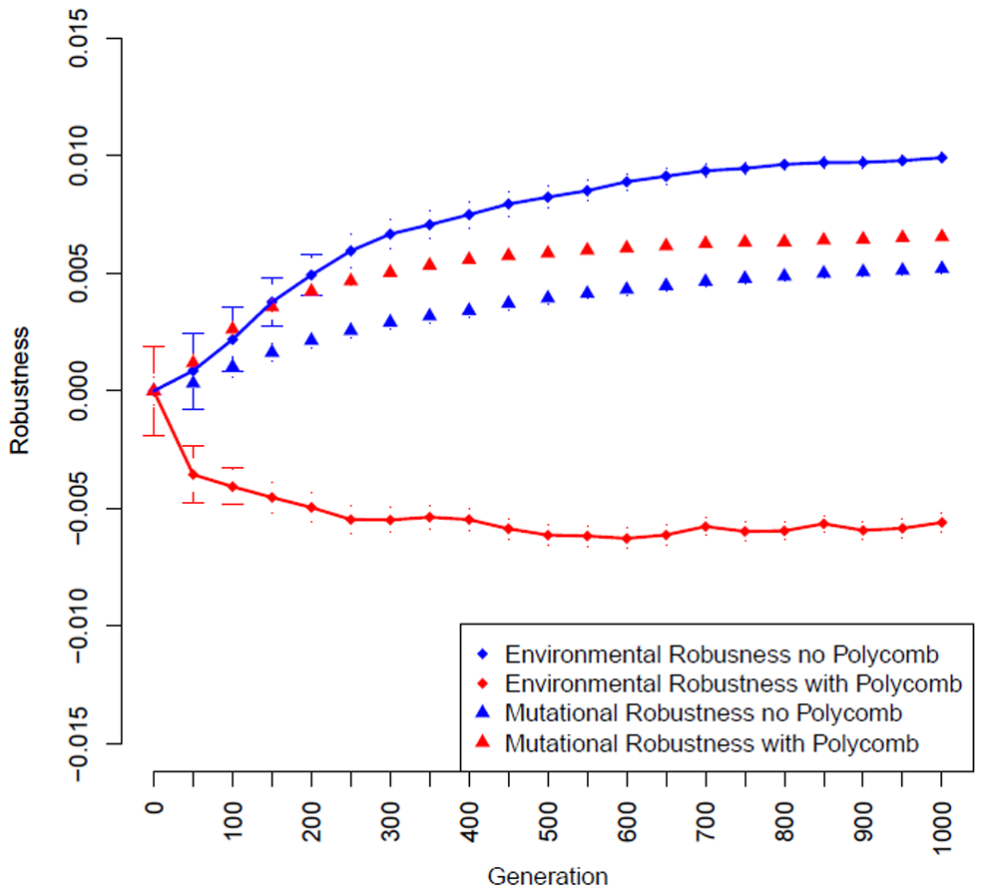
Polycomb functionality decouples environmental and mutational robustness. A gene regulatory network evolving under stabilizing selection without Polycomb, both mutational and environmental robustness increase together over time (blue). With Polycomb the system shows reduced environmental robustness while development of mutational robustness continues (red). All data shown are averages over 200 independent trials using a randomly selected founder individual; error bars represent the SEM, 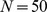, 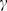 = 0.05, 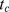 = 2 (see Methods).

In order to assess the impact of the Polycomb mechanism on the evolution of robustness, we measured both environmental and mutational robustness in simulations over 1000 generations. First we set the mutation rate for susceptibility to 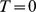 thus eliminating the possibility of evolving any epigenetic function. In keeping with previous results [19] we found that under these conditions both mutational and environmental robustness are positively correlated and increase in tandem (Figure 1, blue lines). However, this relationship was inverted when we allowed the Polycomb mechanism to evolve by setting 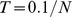 (the same mutation rate per individual used for the matrix 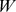 of regulatory interactions). Here mutational robustness increased while environmental robustness decreased (Figure 1, red lines). These results were consistent across a wide variety of parameter values (see Figure S1). In addition, we modeled the results while allowing for a changing network topology (links could be created and destroyed) and found that mutational and environmental robustness remained decoupled see Figure S2). In summary, we have shown that introducing a Polycomb-like epigenetic mechanism into a transcriptional regulation network model is capable of decoupling environmental and mutational robustness.

### Epigenetic functionality allows for the addition of new input/output mappings from a single system

Cell differentiation in multicellular biological organisms usually begins with expression changes in a small number of key differentiation genes in response to environmental cues, often upstream genes in the pathway. Expression of a upstream gene will in turn trigger larger sets of downstream genes that distinctly define each cell type. One of the best understood examples of this is during muscle differentiation where the key gene *MyoD* regulates hundreds of downstream targets [47] including important differentiation factors such as muscle specific creatine kinase (MCK) [48] and muscle acetylcholine receptor (AChR) alpha subunit [49]. In multi-celled organisms that use epigenetic regulation, cell types are further determined by chromatin changes that lock the cell fate. In terms of our model, the early differentially expressed genes can be considered as alternative inputs for our system and the transcription of genes in the differentiated cell can be considered the output. We therefore assume that each input/output mapping (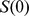
→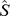) is the equivalent of the cell type and evaluate whether an evolving network is capable of handling multiple input/output mappings(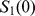
→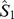, 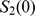
→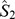 and so on) and in particular whether the capacity to create new mapping is altered by epigenetic functionality in the model.

We therefore allowed a population to evolve under stabilizing selection for 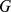 generations (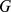 = 100 in main text results; longer values were tested as well. See Figures S1 and S2) and then evaluated whether a randomly selected individual from the population could accommodate a new input state and produce a novel output state (see Methods). The input for the new state was chosen by flipping (0↔1) each binary input with probability 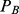 (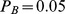 in main text results, though values up to 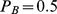 give similar results – see Table S1). The corresponding stable output, 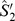, was compared to the initial output, 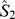, and to the founders initial output, 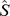, using a normalized distance measures 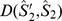 and 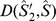 respectively (see Methods) which had to be greater than 0.05 in both cases for 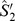 to be considered a new unique output state. If no such significantly different output was found, we repeated the attempt to create a new input/output mapping (random individual, random input state) up to total of 100 times before considering the network unable to create a new input/output state.

**Figure 2:**
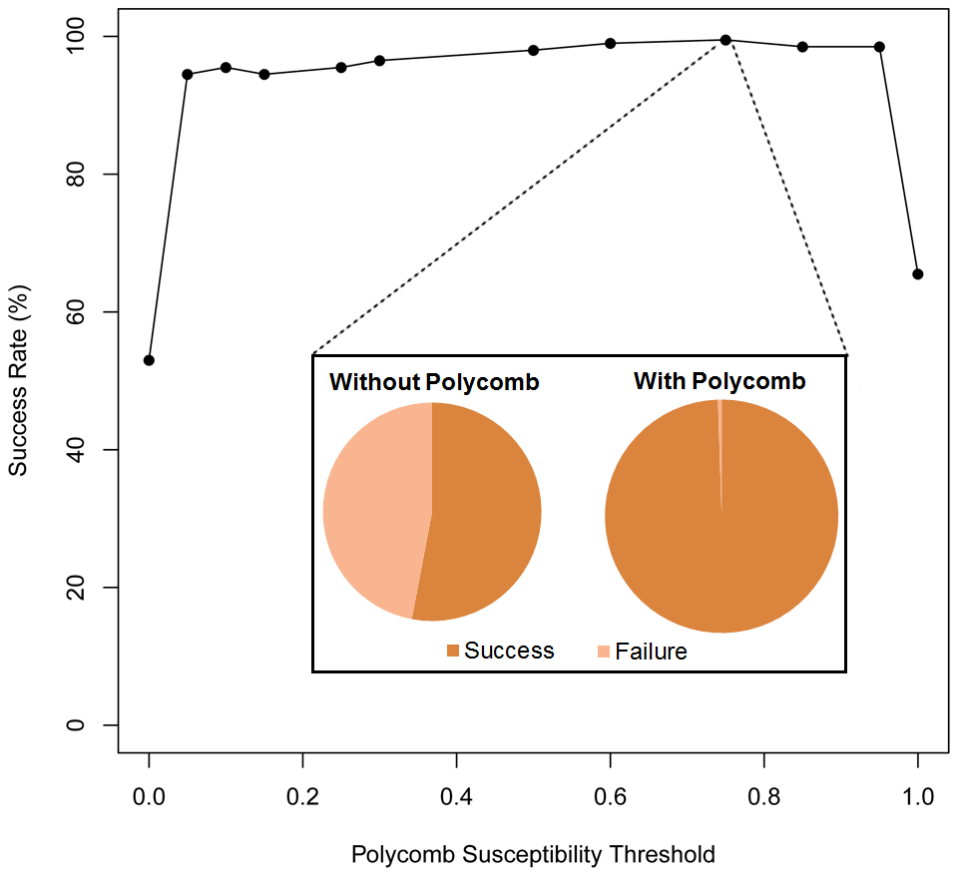
Polycomb functionality promotes the creation of multiple input/output mappings for the same network. In order to determine if a certain expression thresholds are required for the ability to add new input/output mappings we tested the Polycomb threshold (γ) over a wide range of values. When a threshold of 0 is selected no genes are ever be repressed by polycomb (the same as the default cis-regulatory case). At a threshold of 1.0 every gene with a PRE would be repressed, effectively reducing the number of genes in the network. We see the threshold of the Polycomb is not essential for the ability to add new states just the general functionality, N = 200. Inset: With all parameters remaining constant the introduction of polycomb functionality allows the system to add states 99.5% of the time where-as the standard cis-regulatory network is only able to add states 53% of the time. (n = 200, p = 8.62×10^−22^).

Without epigenetic functionality we found that the system was unable to create a new input/output in 47% of 200 cases. However, with Polycomb it was able to find a new input/output 100% of the time (Fig. 2 inset), a highly significant difference (p = 8.62×10^−22^, Fisher's exact test) suggesting that introducing the epigenetic mechanism enabled networks to evolve a strongly increased capacity for adding new input/output states. Multi-stability was found after testing an average of just 7.55 individuals compared to the case without Polycomb where we were unable to detect multistability even after testing 100 individuals. Furthermore, the difference is highly robust to different values in the Polycomb threshold (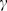) as shown in Figure 2, since starting with values of 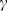 = 0.05 we already have a capacity above 99% of accepting a new state across many parameter values. These results are in accordance with the result described above showing that environmental robustness without Polycomb increases through evolutionary time, making the system less likely to produce a unique output even when inputs are altered. However, with Polycomb the network becomes more sensitive to changes in the environment (represented here by changes in the input vector 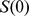) and consequently acquires the capacity for producing a new output when the inputs are perturbed. (In addition, we tested adding multiple new input/output mappings, see SI Table S1).

## Discussion

The role of Polycomb Group Proteins (PcG), discovered in Drosophila, include transcriptional repression of genes showing low expression during early development, a key process in cell differentiation [37]. Homologs of the core functional proteins comprising the PRC-2 complex (a component of PcGs) are present in some eukaryotic unicellar ancestors but are nearly ubiquitous in the multicellular world [39], [40], [41]. The phylogenetic distribution of PcG components and their role in development suggests that Polycomb has played a key role in enabling cell differentiation [40]. In order to study the evolutionary consequences of Polycomb functionality we incorporated Polycomb functionality into a modeling framework [13], [42] which captures key features of gene regulatory networks in an evolutionary context.

The evolution of novel mechanisms for controlling gene expression has evolved in tandem with more complex life forms. Prokaryotes possess *cis*-regulatory elements, operons and some species show evidence of histone style chromatin structure [9]. As the Eukarya evolved from simpler unicellular organisms to complex Metazoa, controlling specialized cell functionality became essential. At the same time, the repertoire of gene expression control expanded to include mechanisms such as methylation, acetylation, ubiquination, and small RNA mediated transcriptional regulation (i.e. RNAi), all of which sculpt gene expression for specialized function [9]. As each of these mechanisms arose, they often functioned “orthogonally” of the others in a mechanistic sense. For example, repression of gene expression can be achieved independently either by cis-regulation (recruitment of repressing TFs to regulatory region) or by histone modifications at the relevant locus. These methods result in the same outcome, transcriptional repression, but work through wholly independent mechanisms.

**Figure 3:**
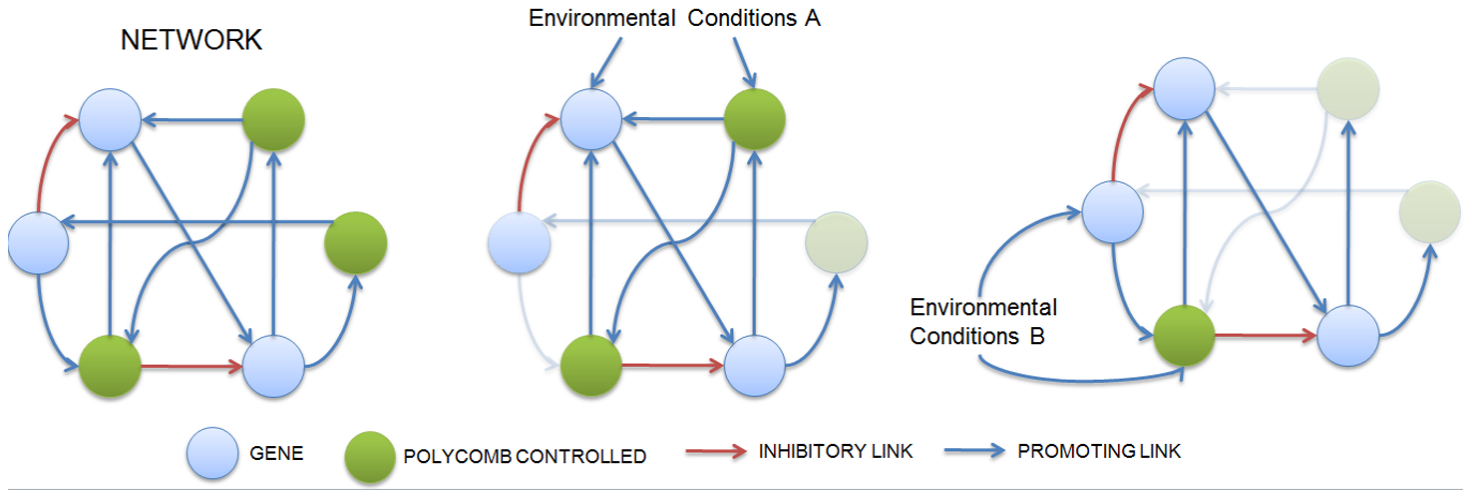
Effective network stratifications by environmental conditions via Polycomb. A) The Polycomb mechanism allows cells to have drastically different phenotypes even though the genotype is identical. Activation of Polycomb during development forces Polycomb-susceptible genes with low levels of expression to be permanently repressed, via chromatin modification, effectively trimming the network (faded interactions). (B and C) Different environmental conditions allow a single network to be trimmed effectively into different networks distinct output states. The final possible outputs of the networks are intimately tied to the original environmental inputs.

By utilizing chromatin states, Polycomb effectively modifies the architecture of the gene regulatory network in real time (Figure 3). As such Polycomb simplifies the architecture by carving out segments of the network to respond to different environmental cues. Polycomb-targeted genes that exhibit low expression during early development (expression of PcGs begins as early as 3 hours post-fertilization in the *Drosophila* embryo) are continuously repressed through heterochromatin formation, nullifying their associated *cis*-regulatory effects. However, under a different set of environmental conditions (i.e., in another developmental context) the same genes might not be enveloped in heterochromatin, allowing the *cis*-regulatory elements to control expression. This method allows cells to use a single set of transcriptional regulators (PcGs) and yet create very different patterns of expression in distinct cell types. For example, undifferentiated mesodermal cells require the expression of *MyoD* to become myoblast cells. However, *MyoD* is repressed through the activity of Polycomb (in particularPRC-2) unless the necessary genes (controlled via adjacency to the PREs) are expressed early in cell division [38]. In this manner Polycomb inhibits *MyoD* in all cells except those destined to become myoblast cells. This design pattern effectively stratifies a single network into many networks, suggesting a functional role for Polycomb in the evolution of cell differentiation, a key requirement for the evolution of multicellularity.

To explore the development of differential expression we evaluated the capacity of the model to accommodate multiple input-output mappings, as in previous studies [50]. We found the ability to adopt multiple input/outputs is greatly facilitated with the functionality of Polycomb (Figure 3). This finding is consistent with the evolutionary data showing that the essential components of Polycomb function are almost ubiquitous in the multicellular world but are rarely all present simultaneously in unicellular organisms [39], [40], [41] again strengthening the hypothesis that Polycomb played a key role during the evolution of multicellularity [3], [4]. Further evidence arises from our finding that evolution under Polycomb decoupled mutational and environmental robustness, suggesting that Polycomb can increase sensitivity to environmental conditions for the purposes of cell differentiation. Previous work has shown that mutational robustness develops in gene-regulatory networks under conditions of stabilizing selection, and that mutational robustness and robustness to environmental changes are correlated [16], [17], [18], [21], [43]. This correlated robustness feature is clearly incongruent with multicellular development where minimal (though particular) environmental cues are capable of drastically changing cellular phenotypes. For example, regulation of only four key transcription factors is needed to change a fibroblast to a cardiomyocyte [26]. When Polycomb functionality is added to the developmental program in the model, this facilitates the effective real-time changes to network connectivity that in turn promotes environmental sensitivity. However, each effective network is still under stabilizing selection so mutational robustness develops. With Polycomb the switch between these effectively distinct network architectures is initiated by changing the initial environmental conditions, making the system more responsive to environmental changes. This real-time remodeling makes use of sub networks for multiple input/output rather than the creation of separate modules within the network. Indeed previous work on the same base model as we used by Borenstein and Krakauer [51] showed that only a limited number of phenotypes of the total phenotype space are possible. It appears that the epigenetic addition to the model makes many of the obtainable phenotypes possible. Biological evidence for decoupling these types of robustness exists in developing multicellular organisms, such as the human fetus, where slight changes in the environmental conditions (for example, exposure to alcohol during the first weeks) can cause severe phenotypic changes [52], [53], indicative of high environmental sensitivity. At the same time, the approximately 70 point mutations acquired on average in each human generation [54] rarely produce catastrophic changes, thus demonstrating high mutational robustness. These findings are consistent with our modeling predictions for a system developing under Polycomb control.

Epigenetic mechanisms have been suggested to evolve in numerous ways. As with the evolution of sexual reproduction, no single explanation has become the definite single explanation for their evolution. Similarly, multicellularity has been suggested to evolve by different means and different mechanisms. Here we put forward an explanation that ties the evolution of multicellularity to that of epigenetic mechanisms. Additionally, we hypothesize that the capacity to respond differently to different environmental signals, as is required during the developmental program of multicellular organisms, is only one evolutionary advantage of epigenetic processes. Other advantages include the contribution of epigenetic mechanisms to the emergence of modularity. It has been argued previously that network modularity contributes to robustness [55]. As we have shown, Polycomb, in response to environmental queues, carves the network into sub-networks such that beyond the critical time only a subset of the interacting elements play a role is shaping the final gene expression pattern. Polycomb, thus, amplifies the effect of environmental perturbation beyond genetic perturbation, and introduces modification at the architectural level. Such change in network architecture introduces higher sensitivity to environmental changes while maintaining robustness to genetic perturbation that have no effect on network architecture. It has been shown that under stabilizing selection, our model tends to decrease the mean number of steps to reach a stable output state [13]. Thus, further analysis of the dependency of time to stable output on the time at which Polycomb is activated (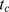
- in our model), would further elucidate the evolutionary role of epigenetic mechanisms.

Metazoan evolution is characterized by specialization of cell and tissue functionality. During multicellular development cells become specialized in function within the organism. This differentiation requires real-time analysis of the local environment to direct cellular development. Our findings, although based on the functionality of Polycomb, suggest a general design principal for evolution in the creation of multicellularity, namely the real-time stratification of the gene network. The effect of the PcG mechanism is to elegantly limit the useable genetic information for a cell based on the events during development. By effectively removing genes from the accessible gene network the complexity of millions of potential interactions among thousands of genes is reduced.

## Methods

### Developmental model

Following Siegal and Bergman [13], the model consists of a gene regulatory network of 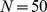 genes each of which has the ability to regulate the expression of any of the 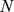 genes. The topology is held in the form of a 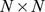 matrix, 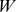 with non-zero entries, *w_ij_*, 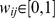 representing connections within the regulatory network (a negative value denotes an inhibitory effect). The non-zero entries in the matrix 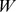 are randomly assigned at the beginning of each simulation with probability 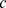 (connectivity of the network). To initiate the development process a random binary (i.e. containing either 0 or 1) initial condition vector 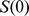 of length 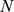 is selected. Gene expression dynamics are then computed according to Equation 1.

Once a stable founder individual is found, a population of a given size (kept constant through the simulation) is founded by that individual. Evolution of the gene network is done through a standard population-genetic process. Mutations occur via changes to the non-zero entries of the matrix with 10% chance of a single mutation per genome. Mating is carried out by selecting two random individuals from the population and then selecting random rows from each parent's matrix to create an offspring genotype (sexual reproduction). At this point selection is carried out as developmental instability (if no equilibrium gene expression can be generated, as calculated by all real components of the eigenvalues of the Jacobian matrix being less than or equal to 0. The Jacobian matrix is defined as: 
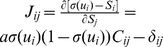
 where 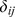 is the Kronecker delta (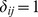 only when 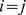, and 0 otherwise) and through distance from an optimal phenotype 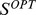 (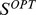 is defined as the 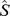 of the initial founder in stabilizing selection) using the formula:
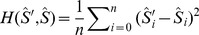
(2)with 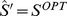


### Robustness

Measuring the mutational robustness of our networks was done in the same manner as multiple previous studies [13], [43], [56]. For each individual in the population we mutated exactly one random connection in the matrix 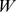. We simulate gene expression dynamics until a new steady state is reached, or until 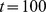, and calculate the phenotypic distance (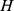) between the new resulting output vector 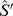 and the original 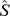 using Equation (2) above.

Identical steady-state 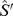 and 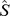 vectors would be considered as having absolute mutational robustness. For sake of clarity we report mutational robustness as 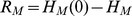. To measure our networks robustness to environmental changes we used a measure outlined in previous studies [43]. In this measure we vary the input vector 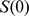 by randomly flipping two members 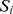 and 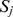 (a 0→1 or 1→0), reflecting the small environmental differences needed to alter cell fate in Metazoa. Using the manipulated input vector 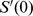 we re-compute gene expression dynamics. After altering the input conditions we calculate the divergence from the original 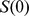 in the same manner as for mutational robustness and report it in the same manner.

## Supporting Information

Figure S1 **Parameter changes and their effect on robustness development.** The model requires fitting of several parameters that can affect the overall outcome of the development of each type of robustness, mutational and environmental. (a) The most important of these parameters is the network size. In the 2002, “Waddington's canalization revisited: developmental stability and evolution” a network of size of 10 is used due to computational limits at the time. Due to increases in computational power our model is able to use a network size of 50 (main text), but we find here that reducing the size does not change the decoupling of mutational and environmental robustness. (b) The slope of the sigmoid function determines how likely genes are pushed to full expression or no expression. (c) The critical period for polycomb to influence gene expression greatly affects the ability of Polycomb to decouple environmental and mutational robustness. If genes have already reached steady state before Polycomb becomes active then environmental robustness will not decrease, as is seen in this example with a high critical time.
(DOCX)

Figure S2
**Robustness evolution with dynamic network architecture.** A regulatory network in which links between nodes could be dynamically created or deleted for each individual in each generation replaced the standard fixed architecture. In this model the rates of adding and deleting connections between nodes are adjusted to keep overall network connectivity stable. The blue points show the evolution of mutational and environmental robustness in the scenario without Polycomb and the red show the scenario with Polycomb. Even when dynamic network architecture is allowed the presence of Polycomb allows for the decoupling of mutational and environmental robustness; 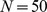, 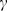 = 0.05, 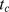 = 2 (see Methods).(DOCX)

Table S1
**Parameter changes and their effect on state addition.** The ability for Polycomb to create a capacity for the network to add new input/output states is unaffected by the different parameters used relating to when and how the new inputs are generated. The number of inputs that are changed to create a new 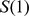 from 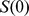 (Perturbation Rate) can be ranged from 5% (figure in main text) to 50% without having any bearing on the ability of Polycomb or the normal condition to add states. The number of generations with stabilizing selection before a new input/output mapping can be changed with no effect on how Polycomb enables the ability to add states. The ability of the normal condition to add states however is diminished after only a few generations. Finally, we can ask the system to add more than 1 new input/output mapping; with Polycomb most networks are able to add more than 1 input. However, without polycomb most networks that were able to add one state are unable to add a second one.(DOCX)
