# Supplementary material for: Epigenetics Decouples Mutational from Environmental Robustness. Did It Also Facilitate Multicellularity?
Source: PLoS Comput Biol. 2014 Mar 6;10(3):e1003450. doi: 10.1371/journal.pcbi.1003450 (PMC3945085; doi:10.1371/journal.pcbi.1003450)

**Figure S1. Parameter Changes ant their effect on robustness development.** The model requires fitting of several parameters that can affect the overall outcome of the development of each type of robustness, mutational and environmental. (a) The most important of these parameters is the network size. In the 2002, “Waddington's canalization revisited: developmental stability and evolution”a network of size of 10 is used due to computational limits at the time. Due to increases in computational power our model is able to use a network size of 50 (main text), but we find here that reducing the size does not change the decoupling of mutational and environmental robustness. (b) The slope of the sigmoid function determines how likely genes are pushed to full expression or no expression. (c) The critical period for polycomb to influence gene expression greatly affects the ability of Polycomb to decouple environmental and mutational robustness. If genes have already reached steady state before Polycomb becomes active then environmental robustness will not decrease, as is seen in this example with a high critical time.

a)


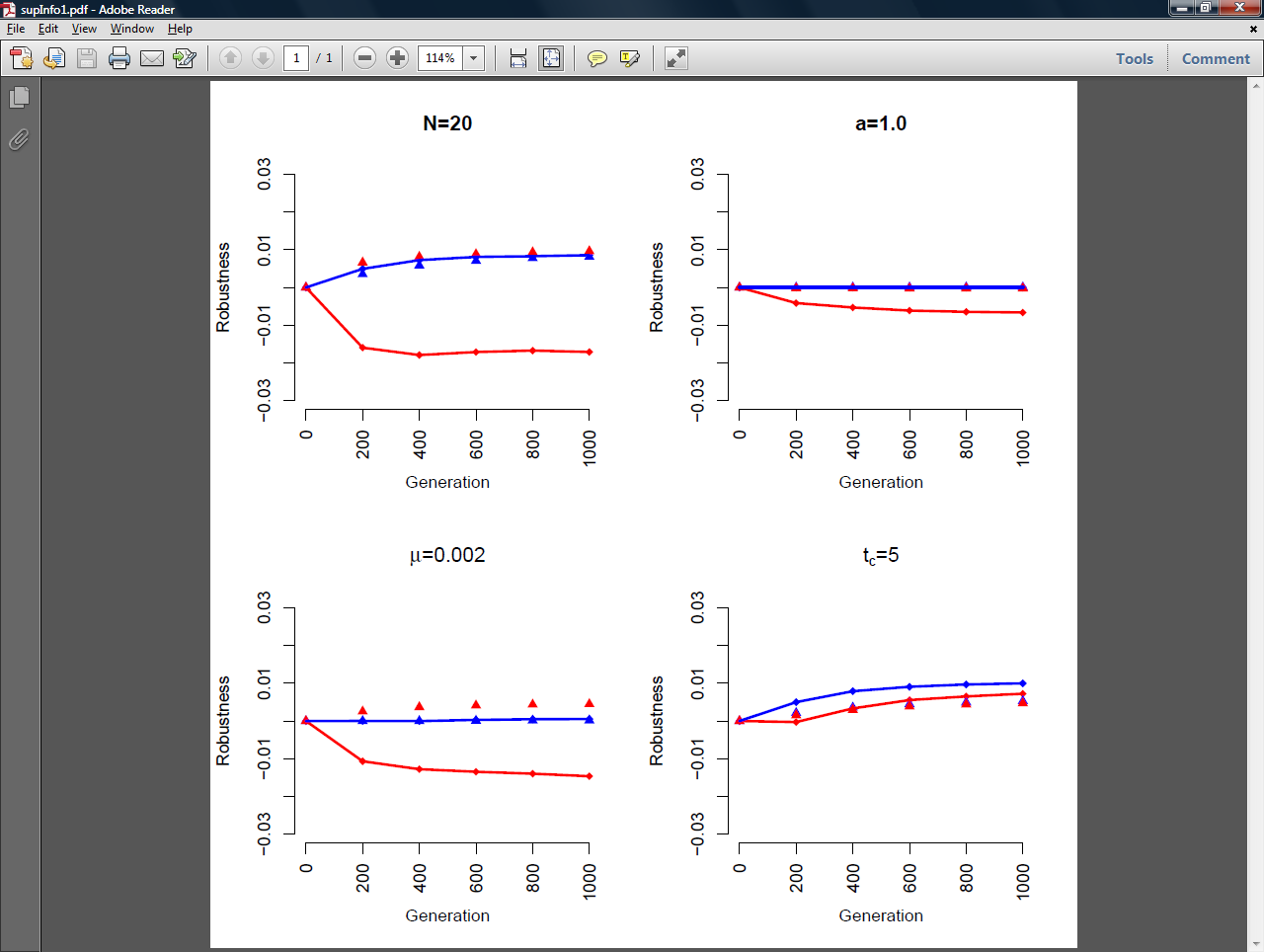


b)


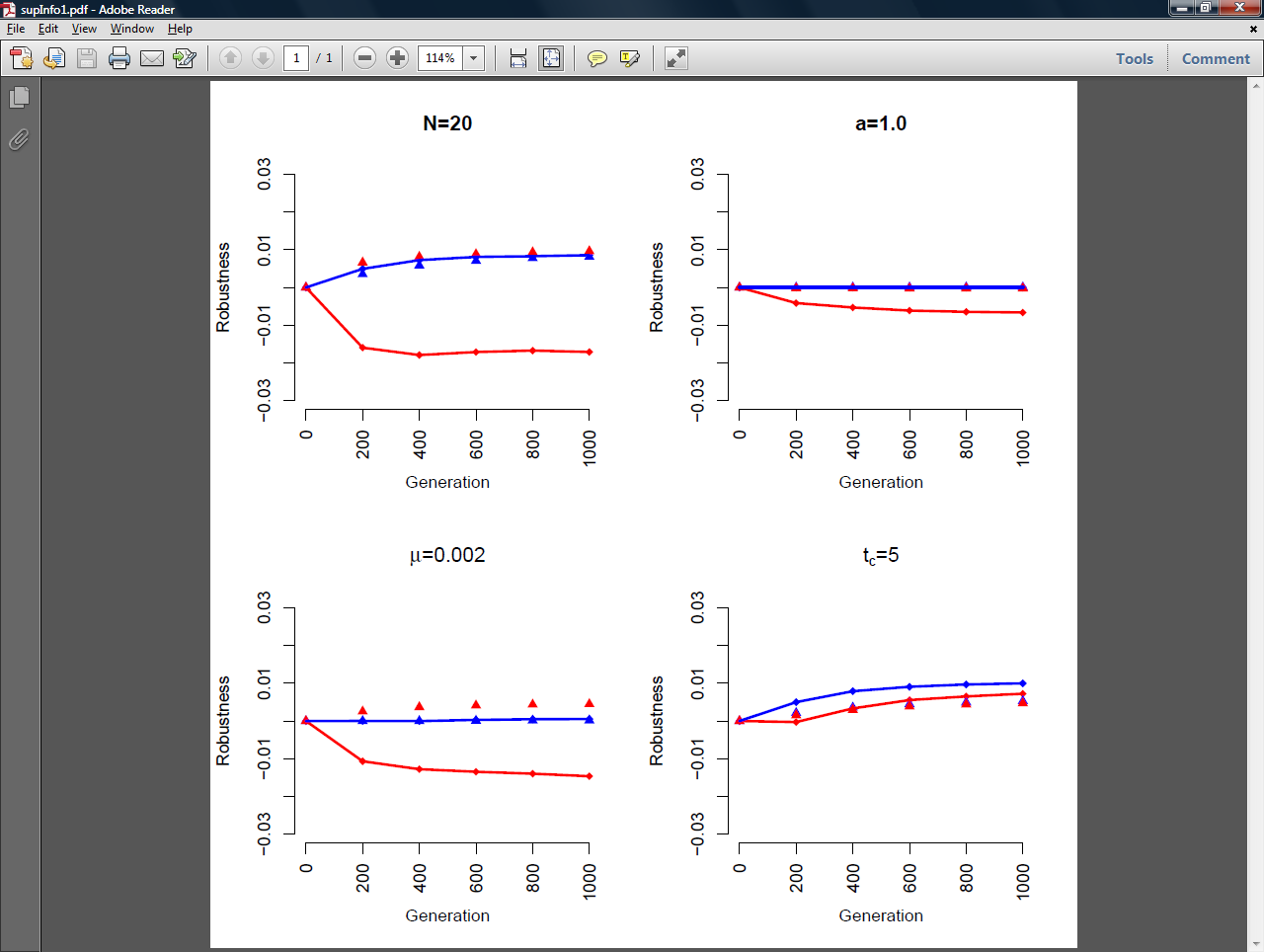


c)


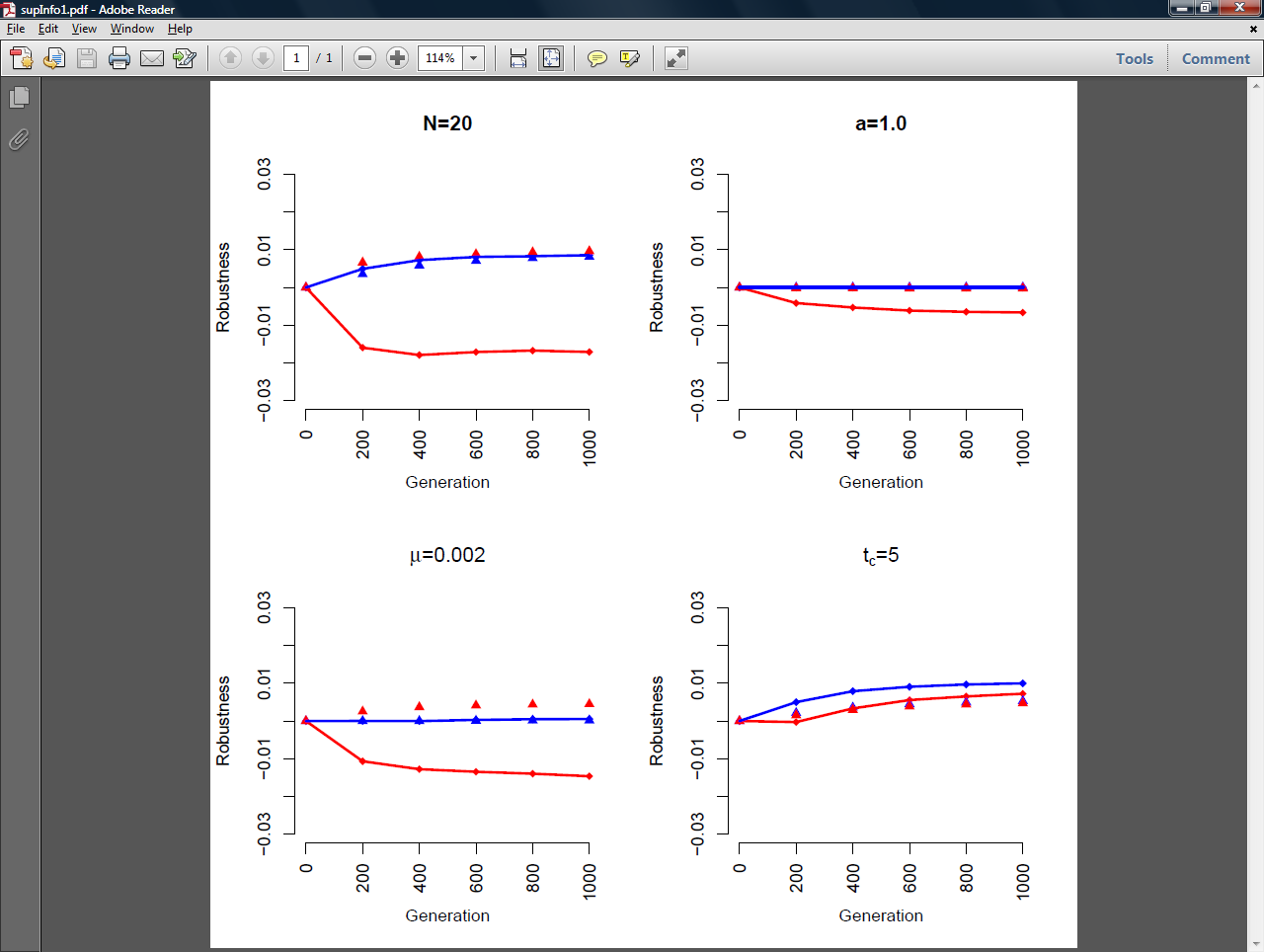


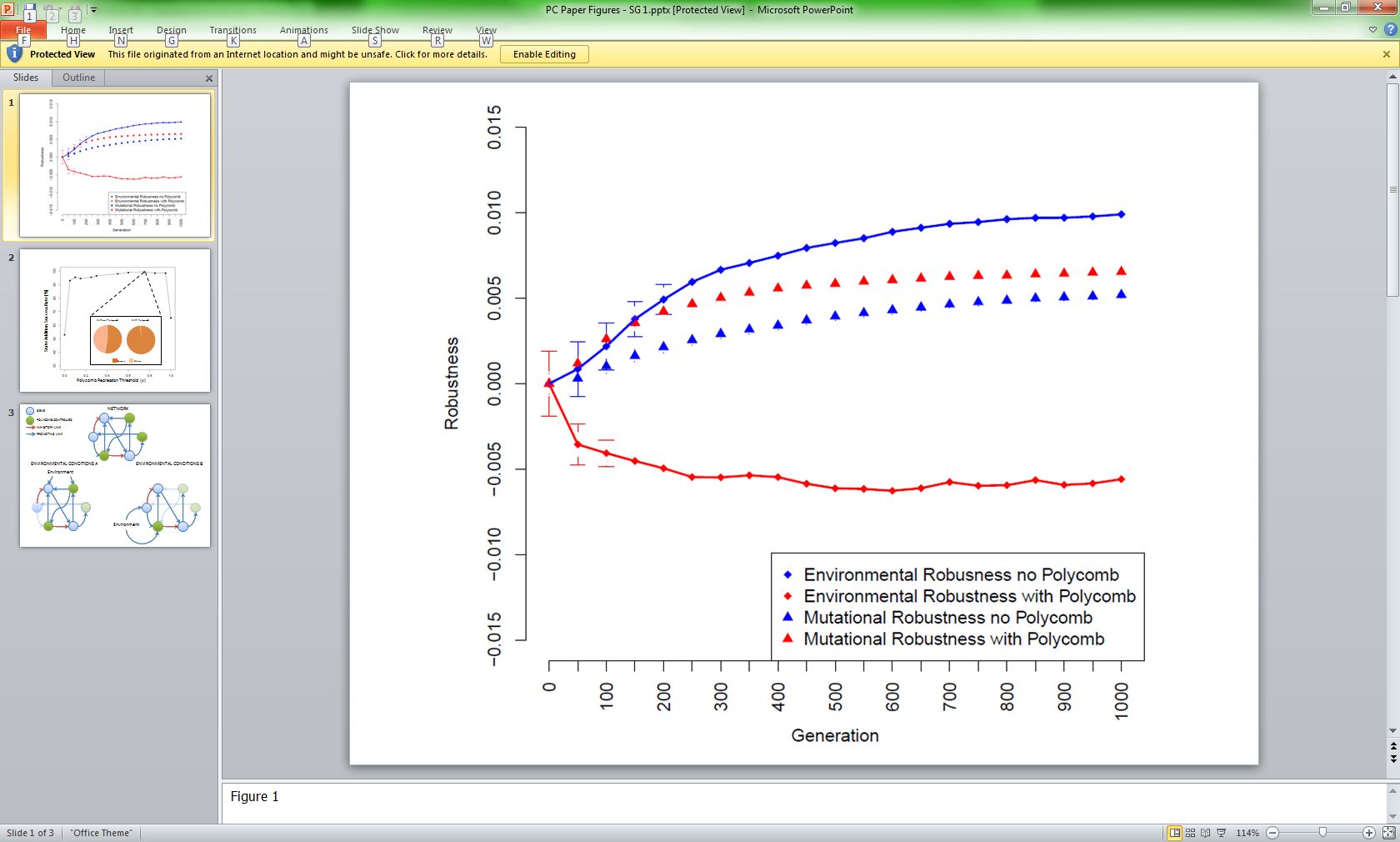

Supplement: Figure S1 — Parameter changes and their effect on robustness development. The model requires fitting of several parameters that can affect the overall outcome of the development of each type of robustness, mutational and environmental. (a) The most important of these parameters is the network size. In the 2002, “Waddington's canalization revisited: developmental stability and evolution” a network of size of 10 is used due to computational limits at the time. Due to increases in computational power our model is able to use a network size of 50 (main text), but we find here that reducing the size does not change the decoupling of mutational and environmental robustness. (b) The slope of the sigmoid function determines how likely genes are pushed to full expression or no expression. (c) The critical period for polycomb to influence gene expression greatly affects the ability of Polycomb to decouple environmental and mutational robustness. If genes have already reached steady state before Polycomb becomes active then environmental robustness will not decrease, as is seen in this example with a high critical time. (DOCX) [file pcbi.1003450.s001.docx]
