# Supplementary material for: Epigenetics Decouples Mutational from Environmental Robustness. Did It Also Facilitate Multicellularity?
Source: PLoS Comput Biol. 2014 Mar 6;10(3):e1003450. doi: 10.1371/journal.pcbi.1003450 (PMC3945085; doi:10.1371/journal.pcbi.1003450)

**Figure S2. Robustness evolution with dynamic network architecture.** A regulatory network in which links between nodes could be dynamically created or deleted for each individual in each generation replaced the standard fixed architecture. In this model the rates of adding and deleting connections between nodes are adjusted to keep overall network connectivity stable. The blue points show the evolution of mutational and environmental robustness in the scenario without Polycomb and the red show the scenario with Polycomb. Even when dynamic network architecture is allowed the presence of Polycomb allows for the decoupling of mutational and environmental robustness;$N=50$,$\gamma$=0.05 , $t_{c}$=2 (see Methods).


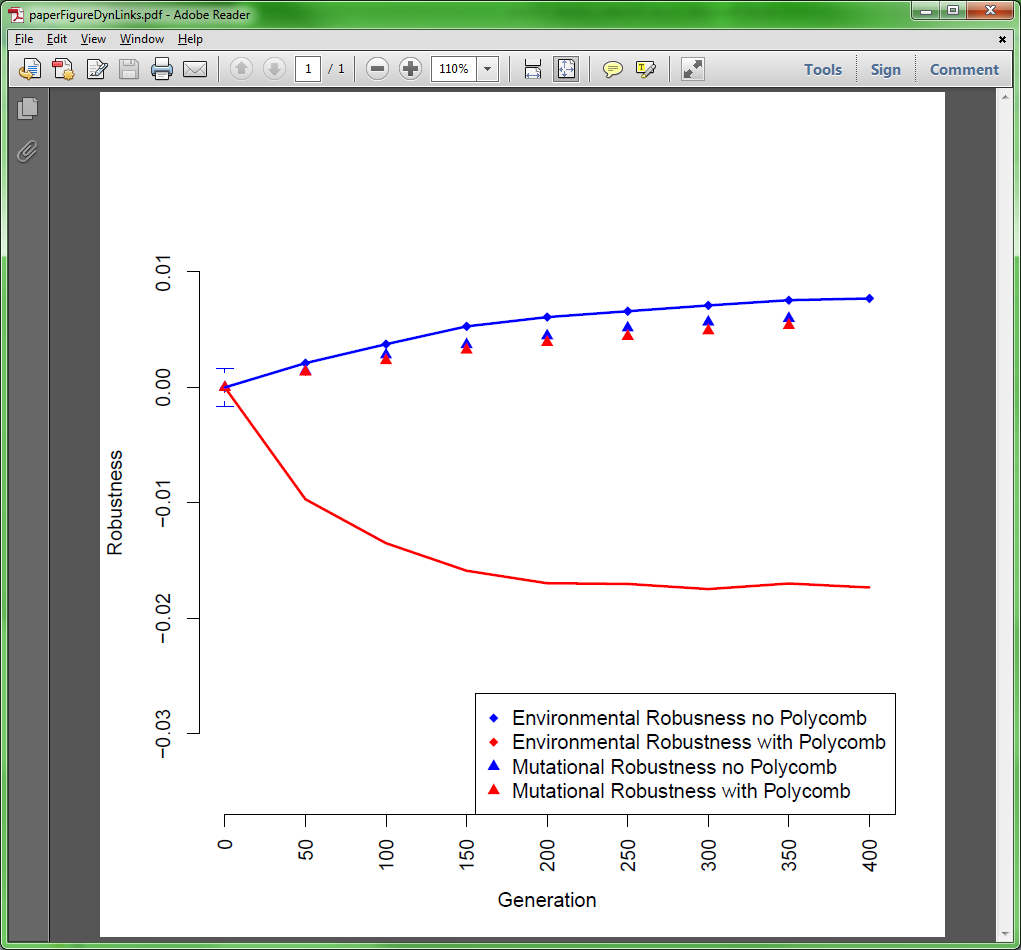

Supplement: Figure S2 — Robustness evolution with dynamic network architecture. A regulatory network in which links between nodes could be dynamically created or deleted for each individual in each generation replaced the standard fixed architecture. In this model the rates of adding and deleting connections between nodes are adjusted to keep overall network connectivity stable. The blue points show the evolution of mutational and environmental robustness in the scenario without Polycomb and the red show the scenario with Polycomb. Even when dynamic network architecture is allowed the presence of Polycomb allows for the decoupling of mutational and environmental robustness; , = 0.05, = 2 (see Methods). (DOCX) [file pcbi.1003450.s002.docx]
