# Supplementary material for: Epigenetics Decouples Mutational from Environmental Robustness. Did It Also Facilitate Multicellularity?
Source: PLoS Comput Biol. 2014 Mar 6;10(3):e1003450. doi: 10.1371/journal.pcbi.1003450 (PMC3945085; doi:10.1371/journal.pcbi.1003450)
Supplement: Table S1 — Parameter changes and their effect on state addition. The ability for Polycomb to create a capacity for the network to add new input/output states is unaffected by the different parameters used relating to when and how the new inputs are generated. The number of inputs that are changed to create a new from (Perturbation Rate) can be ranged from 5% (figure in main text) to 50% without having any bearing on the ability of Polycomb or the normal condition to add states. The number of generations with stabilizing selection before a new input/output mapping can be changed with no effect on how Polycomb enables the ability to add states. The ability of the normal condition to add states however is diminished after only a few generations. Finally, we can ask the system to add more than 1 new input/output mapping; with Polycomb most networks are able to add more than 1 input. However, without polycomb most networks that were able to add one state are unable to add a second one. (DOCX) [file pcbi.1003450.s003.docx]

**Table S1. Parameter changes and their effect on state addition.** The ability for Polycomb to create a capacity for the network to add new input/output states is unaffected by the different parameters used relating to when and how the new inputs are generated. The number of inputs that are changed to create a new $S(1)$ from $S(0)$ (Perturbation Rate) can be ranged from 5% (figure in main text) to 50% without having any bearing on the ability of Polycomb or the normal condition to add states. The number of generations with stabilizing selection before a new input/output mapping can be changed with no effect on how Polycomb enables the ability to add states. The ability of the normal condition to add states however is diminished after only a few generations. Finally, we can ask the system to add more than 1 new input/output mapping; with Polycomb most networks are able to add more than 1 input. However, without polycomb most networks that were able to add one state are unable to add a second one.

| Condition | Polycomb | Normal |
| --- | --- | --- |
| Perturbation Rate = 0.05* | 99.5% | 53.0% |
| Perturbation Rate = 0.1 | 100.0% | 64.5% |
| Perturbation Rate = 0.25 | 100.0% | 69.5% |
| Perturbation Rate = 0.5 | 100.0% | 73.0% |
| Generation for Insert = 50 | 94.0% | 46.0% |
| Generation for Insert = 100* | 99.5% | 53.0% |
| Generation for Insert = 200 | 100.0% | 61.0% |
| Generation for Insert = 400 | 100.0% | 55.0% |
| Num additional States = 1* | 99.5% | 53.0% |
| Num additional States = 2 | 94.5% | 25.0% |
